# Supplementary material for: Radioiodine Exhalation Following Oral I-131 Administration in a Mouse Model
Source: Biomedicines. 2025 Apr 8;13(4):897. doi: 10.3390/biomedicines13040897 (PMC12025149; doi:10.3390/biomedicines13040897)
Supplement: Supplementary file 1 [file biomedicines-13-00897-s001.zip › biomedicines-3456247-supplementary.pdf]

Supplemental Data

Table S1: Pre-application of KI, oral administration of 0.1 MBq and 10 MBq I-131 (Figure 5)

Pre-application of **KI**, oral administration of **0.1 MBq of I-131**

	Controls			0.01 mg KI			0.1 mg KI			1 mg KI		
	Mean	SD	N	Mean	SD	N	Mean	SD	N	Mean	SD	N
Aerosol [%]	18.2	4.8	6	19.4	7.9	6	21.8	7.8	5	15.4	2.4	6
I_2 [%]	8.2	4.7	6	23	3.8	6	24.4	7.5	5	53.7	7.9	6
org. I-131 [%]	73.7	2.3	6	57.7	9.3	6	53.7	7.9	5	28	6.4	6

Pre-application of **KI**, oral administration of **10 MBq I-131**

	Controls			0.01 mg KI			0.1 mg			1 mg KI		
	Mean	SD	N	Mean	SD	N	Mean	SD	N	Mean	SD	N
Aerosol [%]	17,044	6	6	16,9	3,5	4	8,6	7,7	5	13,3	2,3	4
I_2 [%]	15,56	4,5	6	39,5	4,6	4	68,5	17,2	5	58,3	6,8	4
org. I-131 [%]	66,8	5,7	6	43,6	7,9	4	22,8	15,6	5	28,5	4,9	4

Table S2: Pre-application of $KClO_4$, oral administration of 0.1 MBq and 10 MBq I-131 (Figure 6)

Pre-application of **$KClO_4$**, oral administration of **0.1 MBq I-131**

	Controls			0.05 mg perchlorate			1 mg perchlorate			5 mg perchlorate		
	Mean	SD	N	Mean	SD	N	Mean	SD	N	Mean	SD	N
Aerosol [%]	18.2	4.8	8	18.4	4	8	23.4	11.6	8	21.4	16.6	7
I_2 [%]	8.2	4.7	8	19.5	9.7	8	34	7.1	8	29.1	7.2	7
org. I-131 [%]	73.7	2.3	8	62.1	8.5	8	42.6	6.8	8	49.5	17.7	7

Pre-application of **$KClO_4$**, oral administration of **10 MBq I-131**

	Controls			0.05 mg perchlorate			1 mg perchlorate			5 mg perchlorate		
	Mean	SD	N	Mean	SD	N	Mean	SD	N	Mean	SD	N
Aerosol [%]	17,044	6	6	13,4	4,5	6	14,5	2,8	6	8,3	3,2	6
I_2 [%]	15,56	4,5	6	27,8	6	6	30,8	7,6	6	40,1	13,5	6
org. I-131 [%]	66,8	5,7	6	58,9	9,2	6	54,7	9,5	6	51,7	10,5	6

Table S3: Pre-application of carbimazole and L-thyroxine, 0.1 MBq and 10 MBq I-131 (Figure 7)

Pre-application of **carbimazole and L-thyroxine**, oral administration of **0.1 MBq I-131**

	Controls			Carbimazole			L-Thyroxine		
	Mean	SD	N	Mean	SD	N	Mean	SD	N
Aerosol [%]	18,2	4,8	8	18,4	4	8	23,4	11,6	8
I_2 [%]	8,2	4,7	8	19,5	9,7	8	34	7,1	8
org. I-131 [%]	73,7	2,3	8	62,1	8,5	8	42,6	6,8	8

Pre-application of **carbimazole and L-thyroxine**, oral administration of **10 MBq I-131**

	Controls			Carbimazole			L-Thyroxine		
	Mean	SD	N	Mean	SD	N	Mean	SD	N
Aerosol [%]	17,044	6	6	28,3	5,6	6	43,5	11,1	6
I_2 [%]	15,56	4,5	6	21,8	9,6	6	24,8	7,1	6
org. I-131 [%]	66,8	5,7	6	49,9	8,9	6	31,7	11,2	6
